# Restitution Through Equity-Focused Mentoring: A Solution to Diversify the Physician Workforce

**DOI:** 10.3389/fpubh.2022.879181

**Published:** 2022-06-01

**Authors:** Valencia P. Walker, Dominique R. Williams

**Affiliations:** ^1^Department of Pediatrics, Nationwide Children's Hospital, The Ohio State University College of Medicine, Columbus, OH, United States; ^2^Center for the Study of Racism, Social Justice and Health, Fielding School of Public Health, University of California, Los Angeles (UCLA), Los Angeles, CA, United States; ^3^Division of Primary Care, Center for Healthy Weight and Nutrition, Nationwide Children's Hospital, Columbus, OH, United States

**Keywords:** mentoring, academic medicine, diversity, equity, inclusion

## Abstract

Minoritized and marginalized physicians who identify as Black, Latino/a/x and Native American (BLNA) remain unacceptably underrepresented in medicine. Multiple studies provide a compelling argument for prioritizing racial/ethnic diversification of the physician workforce to improve racial/ethnic physician-patient concordance and assist in achieving more equitable health outcomes. Despite a growing awareness for the tangible benefits of a diversified physician workforce, the number of physicians from minoritized and marginalized groups remains relatively stagnant or worsening in certain demographics. The 5:1 ratio of Black students and trainees to Black faculty exemplifies and exacerbates the increased risk for harmful isolation particularly experienced by many BLNA mentees. They too need and deserve the benefits produced by concordant racial/ethnic faculty mentoring and support. However, these demands on time, resources and bandwidth can lead to negative consequences for BLNA faculty engaged in these efforts by contributing to their emotional, mental and physical exhaustion. Given the perpetual paucity of BLNA physicians in academic medicine, immediate interventions to prevent attrition of BLNA faculty, trainees and students journeying along the physician career pathway are urgently needed. Requiring the implementation of mentoring programs explicitly focused on increasing the number of physicians from groups underrepresented in medicine must happen at every point of the education and training process.

## Introduction

The Association of American Medical Colleges (AAMC) defines “underrepresented in medicine” (URiM) as “…those racial and ethnic populations that are underrepresented in the medical profession relative to their numbers in the general population” (1). This definition lacks an explanation for “underrepresentation.” Historically, minoritized and marginalized physicians who identify as Black, Latino/a/x and Native American (BLNA) are unacceptably underrepresented in medicine, and the AAMC definition of URiM fails to ascribe any cause to the observed discrepancies. Examining the lack of representation reveals a myriad of past, present and continuing programs and practices that perpetuate a system structured to produce unequal, unjust and inequitable outcomes. Despite an increasing awareness for the tangible benefits of a diversified physician workforce, the number of physicians from minoritized and marginalized groups is stagnant or worsening in certain demographics (2, 3). A growing body of literature provides evidence for increasing racial/ethnic physician-patient concordance to attain more equitable health outcomes (4–6). The powerful indictment of failing to assure optimal outcomes for all patients must compel the medical profession to ameliorate this phenomenon of “underrepresentation.”

### Persistence of Racism

Countless barriers contribute to the lack of workforce diversity. Structural racism, however, acts a significant driver of this problem. While frequently described as a resolved issue for the United States, examples of racism and racist actions continue to proliferate in the first two decades of the 21st century (7). The response to Hurricane Katrina in New Orleans (8). The “cost-saving” impetus for the water crisis in Flint, Michigan (9, 10). The death of Ms. Sandra Bland while in the custody of Texas police (11, 12). In South Carolina, church executions by a white supremacist (13). Building an access pipeline in South Dakota on territory of Indigenous people (14). Arson of three Black churches in Louisiana by a young white man who admitted to being influenced by rhetoric of a far-right musician (15). Mr. Ahmaud Arbery murdered by three white men for jogging in their south Georgia neighborhood (16). Punitive immigration policies that separate children and their families. (17). Onlookers watching for more than eight and a half minutes as Mr. George Floyd, pinned to the ground by a Minneapolis police officer, cried out “I can't breathe” more than 20 times before he died (18). Each representing a horrific event in isolation, but structural racism acting as the common thread binding these incidents together.

Amid these repeated acts of racialized violence and terror, BLNA faculty and trainees carry on with the demands of their roles as if none of it creates anything amiss in their lives. They continue to care for patients. They keep studying for exams that determine competency in their respective specialties. Meeting these required expectations does not imply that they are impervious to the heaviness of heartaches felt in their communities and portrayed by the news. The biases of others that draw greater scrutiny and criticism of their intellectual abilities and their clinical acumen add insult to the injuries. To persevere, they attempt to reconcile their membership in a profession that simultaneously provides gratifying purpose and expects them to diminish or outright dismiss the detrimental effects of social injustices and structural racism all around them. They persevere by learning and practicing in academic and sociopolitical climates that promulgate chaos and disruption in their lives.

### Racism and Social Frameworks

**Figure 1:**
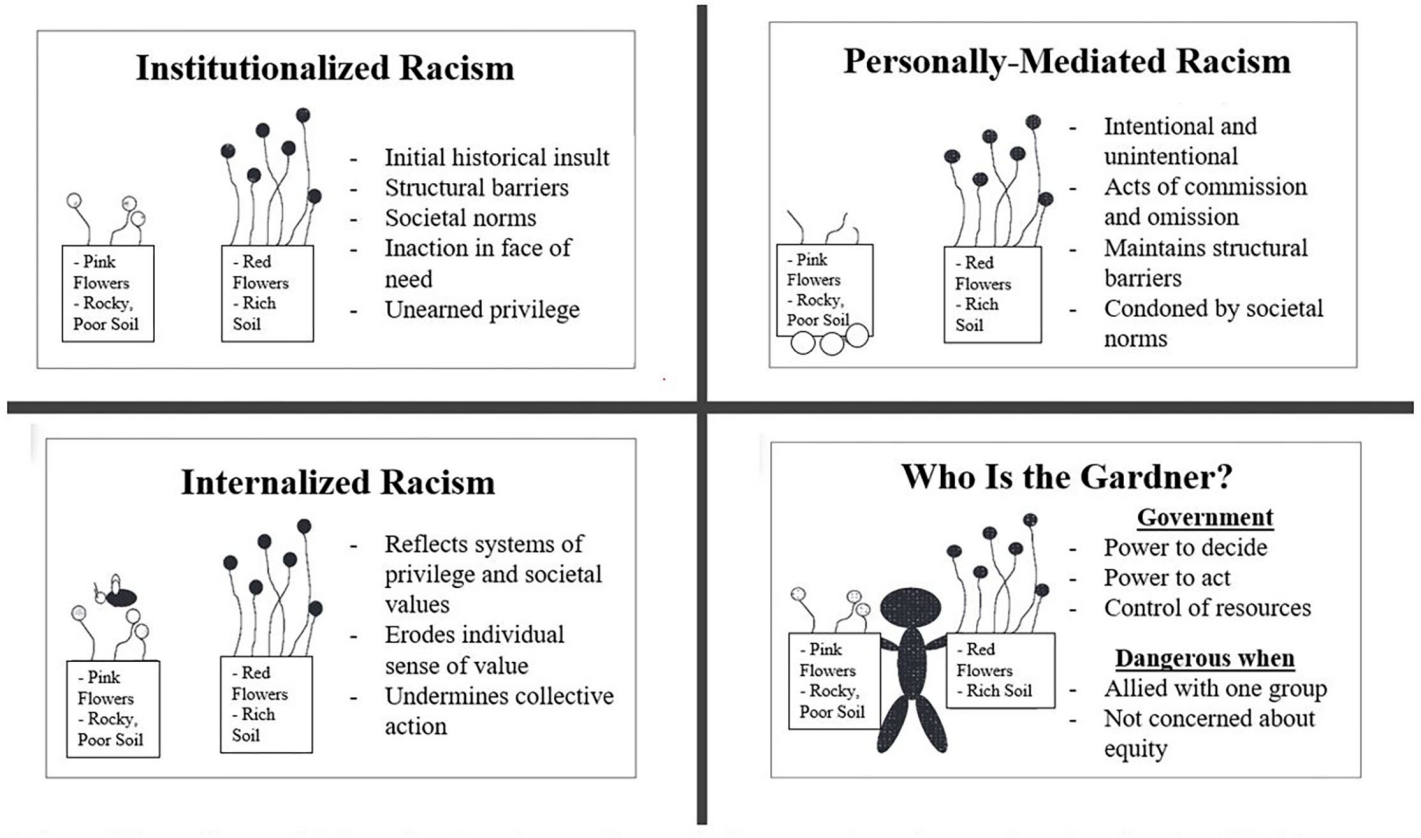
Adaptation of the Gardener's Tale. Adapted from Jones (20).

In the 1960s, Stokely Carmichael introduced the term institutionalized racism. In an excerpt from his piece in The Massachusetts Review, Carmichael writes “When unidentified white terrorists bomb a Negro church and kill five children, that is an act of individual racism, widely deplored by most segments of the society. But when in that same city, Birmingham, Alabama, not five but five hundred Negro babies die each year because of a lack of proper food, shelter and medical facilities, and thousands more are destroyed and maimed physically, emotionally and intellectually because of conditions of poverty and deprivation in the ghetto, that is a function of institutionalized racism” (19). In 2000, Dr. Camara Phyllis Jones published “The Gardener's Tale,” an allegory of a gardener with two flower boxes of different colored flowers (20). Her allegory illustrated the different levels of racism working within society (Figure 1). The gardener develops a preference for the red flowers growing in the rich soil, convinced that red flowers were intrinsically better than pink ones, all while doing nothing to improve the poor, rocky soil in which the pink flowers attempted to grow. Dr. Jones used her allegory to explain how institutionalized racism generated associations between socioeconomic status and race through the discriminatory practices that created racialized differences in access to education, housing, employment, and voting rights. Subsequently, these factors adversely affect healthcare and health outcomes, in part, by derailing efforts to build and sustain pathways to professional development for the medical profession.

### Racism in Medicine

Historically, the medical profession has perpetuated racialized biases and racist practices through its established systems, policies and hierarchies of power. Written in 1910, the Flexner Report received acclaim for revolutionizing medical education (21). Its recommendations also devalued Black people and further perpetuated racial biases. The author, Abraham Flexner, maintained that Black physicians were assigned to “keep [African-Americans] healthy enough not to contaminate nearby White people” (22). In his view, historically Black medical schools should focus on hygiene for Black people, rather than introducing students to surgery and comprehensive medical care for their patients. That type of training was reserved for white people. This widened the racial chasm in medicine and led to the closure of all but two of the seven Black medical schools operating at that time (23). Although the Carnegie Foundation sponsored the report and supported its findings, the Foundation effectively turned its back on the two remaining Black medical schools—Meharry and Howard—when they sought funding to help implement the [costly] recommendations from the Flexner Report. In response to their requests, the Foundation replied, “If we start helping medical colleges for colored people we cannot discontinue.” (24).

As a consequence, people from minoritized and marginalized communities disproportionately experienced disenfranchisement and health inequities. Early medical professionals asserted that Black patients had “natural immunity” to certain diseases or manifested diseases differently because of their race. These notions provided the backdrop for spreading misinformation during the outbreak of yellow fever in Philadelphia and were the basis for the Tuskegee Study of Untreated Syphilis in the Negro Male, one of the longest, non-therapeutic experiments in medical history (25, 26). Over the course of 40 years (1932–1973), the study observed 400 men to document any “racial differences in clinical manifestations of syphilis” (26).

In the 1950s and 1960s, the forced sterilization of Puerto Rican women occurred due to a U.S. government sanctioned mass-sterilization policy (27). More than historical footnotes, the racist beliefs that drove these and other justifications for mistreatment of BLNA people persist in science and medicine. Ignorance and inaccurate presumptions about racial differences influence current clinical practice, institutional culture and medical education curricula. For example, racialized disparities in pain assessment and management are thought to be deeply rooted in myths that Black people have higher tolerance for pain than white people because of their “thicker skin” (28, 29). Despite alarming inequities in health outcomes for Indigenous people, there are persistent disparities in personal health care expenditures by Indian Health Services compared to U.S. National Healthcare expenditures (30).

In 2020, the American Academy of Pediatrics (AAP) issued an apology for its history of repeatedly barring the acceptance of Black physicians as members and supporting concessions of their membership due to race (31). The experiences of Drs. deGrate Smith and Scott also included exclusion from their local chapter of the American Medical Association (AMA). Without membership or certification from the AMA, Drs. deGrate Smith, Scott and countless others were unable to care for their patients without disruption; neither could they contribute to the medical policies that affected their patients (31). The longstanding history of segregation and exclusion within the AMA was one of many reasons for the founding of the National Medical Association, the nation's largest and oldest organizations dedicated to African American physicians and their patients since the late 19th century (32). About 75 years later, the Association of American Indian Physicians formed to address the ongoing health disparities faced by Native American communities (33). The formation of the National Hispanic Medical Association in 1994 demonstrated the ongoing need for minoritized and marginalized physicians to advocate for health equity within their communities (34).

### Diversifying Academic Medicine

When considering the historical and ongoing barriers to increasing the number of physicians underrepresented in medicine, the 5:1 ratio of Black trainees to Black faculty exemplifies the amount of work necessary to dismantle structural racism in medicine that remains undone (35). Statements of solidarity alone cannot translate into tangible investments toward solutions for these long-standing issues. Although responding to sociopolitical unrest and multiple crises triggered by racist and divisive rhetoric now appears trendy and short-lived for far too many in the majority, BLNA faculty wrestle with the distress these issues create within and outside of the academic or clinical environment. BLNA mentees experience isolation and lack of support. They need and deserve the benefits produced by concordant racial/ethnic faculty mentoring. However, emotional, mental and physical exhaustion as a negative consequence of engaging in these efforts can adversely impact academic productivity and advancement among the BLNA faculty, who incorporate an often out-sized proportion of mentoring responsibilities into their professional portfolio. Furthermore, experiences with invalidation by other faculty members can interrupt, mar or derail the careers of BLNA faculty.

BLNA faculty learn to maintain a balance between the despair and the data, the sympathy and the statistics and even the rage and rational. With that clarity, research into evidence-based methods for solutions to the problems of underrepresentation ought to highlight BLNA faculty and amplify their lived experiences within the profession. Leaders of academic medicine need to welcome opportunities to learn how BLNA faculty navigate the labyrinth of shielding the burdens of their emotional vulnerabilities; providing an academic discourse to justify the reality of their experiences; and even proposing data-driven, solutions. Understanding the labyrinth can improve the profession of academic medicine and potentially spark innovations that improve patient care.

Although some accept the concept of structural racism, many struggle with addressing the racism driving racialized mistreatment. Use of racial quotas and affirmative action programs have repeatedly failed to reconcile racialized disparities in progress. The Bakke decision—*Regents of the University of California vs Bakke (1978)*—exemplified the vitriolic backlash against quotas and affirmative action programs benefitting BLNA people (36). This Supreme Court case also exposed deeply entrenched, biased opinions that increasing racial and ethnic representation required medical schools to lower their standards and accept less qualified students (37). The prevailing presumption held that these practices displaced more qualified and worthy white students (37, 38). Almost 45 years later, the biases persist, and schools struggle to differentiate evidence-based holistic review practices from misguided arguments linking affirmative action programs to reverse racism and outdated notions of racial quotas (39–41).

Alternatively, efforts explicitly aimed at restitution begin with acknowledgment and acceptance of the pain and suffering created by racism and endured for decades by minoritized and marginalized communities. Structural racism exists everywhere, including within the medical profession. Implicit white race preference can adversely influence actions to diversify medicine (42). Intentional efforts to mitigate racialized bias; recruit a diverse pool of applicant; and establish evidence-based initiatives for holistic review of applicants hold promise (39, 41, 43, 44). Academic medicine must also heed the ongoing calls to develop anti-racist policies and build a racially/ethnically diverse workforce through optimization of professional pathways. To accomplish these directives requires nurturing an environment that prioritizes inclusion, belonging and equity at its institutions.

Despite comprising almost 15% of the population, only 7% of medical students and trainees are Black. More alarmingly, only 3.6% of full-time medical school faculty are Black (45). From generalists to specialists, over the past few decades, physicians from groups URiM comprise a meager percentage of faculty in their specialties, and growth lags behind the proportion of women in medicine (46–48). Moreover, far fewer URiM faculty achieve the rank of associate or professor (46, 47).

Even more telling is the outsized role of Historically Black College and University (HBCU) medical schools in producing Black physicians. If only two of the other five Black medical schools had remained opened after 1910, an estimated 10,000 additional students would have graduated from them between 1910–2019 (23). Assuming similar rates of growth as Meharry and Howard, the four (theoretical) schools would account for an 11% annual increase in the number of Black medical graduates, instead of the 1% increase currently observed (23). Given the present-day consequences of the Flexner Report and related racialized policies, the need for equity-focused interventions is evident (23, 24).

HBCU medical schools account for 33% of Black chairs, 10% of Black faculty and 14% of Black students (49). When examining the data for representation of people that are of Hispanic, Latino/a/x or Spanish origin, the disparities widen. Despite comprising approximately 18% of the U.S. population, barely 10% of matriculants to medical school and only 5.5% of full-time medical school faculty are Hispanic, Latino/a/x or of Spanish origin (2, 3, 50). The data for the Native American community are abysmal (2, 3, 50, 51).

The AAP acknowledged the need to increase workforce diversity to improve concordance with the increasing racial/ethnic diversity of patients served (52). Among possible solutions, they highlighted the role of sponsorship as an important approach to improve representation and advancement of URiMs in academic medicine (52, 53). However, to date, the AAP lacks formally supported, structured mentoring programs that directly and equitably address workforce diversity. On a broader scale, the NIH's National Research Mentoring Network encourages URiMs to pursue careers in biomedical research. The program provides networking, professional development and mentoring activities to address long-standing racial/ethnic inequities in NIH grant funding (54). Yet, it continues to struggle with reversing the lack of racial/ethnic diversity among NIH grant recipients.

## Discussion

Non-BLNA faculty ought to know about appropriate resources and initiatives within their respective specialties. Further]more, non-BLNA faculty can take action by signing up as sponsors and champions to help support the viability of such programs. They can facilitate the mentoring that occurs within programs, particularly for initiatives that exist at their local institutions. Most importantly, non-BLNA faculty need to embrace a willingness to live with an unshakeable discomfort that parallels the daily distresses plaguing many of their BLNA colleagues. This includes accepting that these few steps are merely an initial turn toward a positive direction and still cannot address the calamities before us.

To protect against catastrophic loss from an already treacherous pathway into the profession, academic medicine must change course and intentionally set goals informed by equity and justice frameworks. The labor required to accomplish these goals demands proper prioritization and support. Restitution begins with the creation of mechanisms for everyone to hear and respond to the unique concerns of BLNA people navigating academic medicine. Establishing inclusive environments that assure the equitable success of all students and trainees is undeniably past due. Knowing that the unacceptably limited number of BLNA faculty cannot shoulder this enormous responsibility alone, the onus falls on allies and institutions to deliver on the incontrovertible imperative for an immediate response. This happens while simultaneously developing policies and programs that significantly minimize the current gaps in unmet needs.

One of the first challenges for academic medicine involves rejecting the prior status quo and arriving at a place of acceptance, particularly for structural competence (55). Providing a framework to understand the context and history of structural racism situates its role in health disparities. This becomes a foundational component necessary to moving forward with plans to decrease existing disparities and inequities. Investment in structural competence training helps to facilitate a more informed lens in caring for patients and working with students and colleagues. Mentoring relationships can help trainees and colleagues process the ambiguity and discomfort that comes from moving on from the status quo. Appropriately supported and valued BLNA mentors can supply more accessible and tangible examples of how structural competence affects their work with patients. Structural competence training represents a specific example of actions that move beyond statements of solidarity against racism and injustice. It primes institutions and systems to make concrete investments into solutions that remediate these long-standing issues.

Reframing the language and lexicon used by the profession further supports efforts to establish equity-focused mentoring practices. A well-informed professional recognizes the inaccuracy of depicting racial health disparities as a causal relationship, i.e., most Black patients have Type 2 diabetes or hypertension. Instead, the competent physician acknowledges the ever-present components of structural racism, i.e., minoritized groups are disproportionately affected by chronic diseases due to multiple social and environmental factors. Justice-focused curricula teach learners to identify and discuss the interplay of these factors. When the inevitable discomfort or disagreements emerge, an expectation must exist for listening first followed by leaning in with empathy and curiosity. Asking more questions and listening more attentively are responsibilities that educators and leaders can never abdicate (56). These simple actions provide critical support to learners from historically underrepresented and marginalized groups at all stages of their training, and also represent improved practices (57, 58).

Given the perpetual paucity of BLNA physicians in academic medicine, immediate interventions to prevent attrition of BLNA faculty, trainees and students journeying along the physician career pathway are urgently needed. Requiring implementation of mentoring programs explicitly focused on increasing the number of physicians from URiM groups must happen at every point of the education and training process (59). Generational funding commitments (i.e., 10- to 15-year), accompanied by restricted resource allocation, are vital to assuring any reasonable expectations for sustainable success of URiM-focused mentoring programs. These characteristics form a foundation for an equity-focused approach to mentoring. Table 1.

**Table 1:** Interventions to support equity-focused mentoring.

**Interventions to Support Equity-Focused Mentoring**		
**Institutional**	- **Create mechanisms** for documenting and responding to first-hand narratives about the specific concerns and barriers for BLNA faculty in academic medicine - **Direct explicit efforts** to establish and maintain inclusive environments that promote equitable success of all faculty - **Prioritize strategic planning** that institutionalizes programs addressing mentoring and sponsorship support of BLNA faculty - **Support pathways** to promotion and tenure with recognized valuation of mentoring and other educational activities advancing diversity, equity, inclusion and belonging	- **Allocate continual funding** for structural competence training of faculty - **Enact “generational funding**” to ensure sustainability of mentoring programs aimed at ameliorating inequities in representation of BLNA faculty - **Designate institutional funding and FTEs** to support faculty engaged in activities that improve racial/ethnic representation within the workforce and mitigate attrition of BLNA faculty, trainees and students - **Incorporate mentoring curricula** and training for faculty that includes tools for dialogue and mentoring across differences
**Interpersonal**	- **Affirm a commitment** to the equitable success of all students and trainees across learning environments, inclusive of departments/division**s** - **Advocate for policies** that facilitate inclusive learning environments - **Champion mentoring programs** that also demonstrate benefit for BLNA populations and offer extended funding to protect their long-term viability	- **Require structural competence** training for all faculty with their annual professional development activities - **Develop and execute research protocols** that obtain disaggregated data and use it responsibly to evaluate the efficacy of mentoring-related interventions for BLNA populations
**Individual**	- **Implement tenets of structural competence** to modify self-identified behaviors as needed - **Endorse anti-racist review and revision** of curricula and other educational efforts - **Learn about specialty-specific mentoring** programs and resources focused on BLNA populations - **Serve as a mentor** within the local institution	- **Incorporate empathy, self-reflection, and curiosity** into teaching methods and evaluations - **Model behaviors** that demonstrate respectful, inclusive and equitable treatment of colleagues, trainees, students, staff and patients

Based on a presumption that meritocratic principles inherently define the medical profession, many may instinctively criticize and decry such a “favored” approach that benefits select groups. A more objective analysis, however, provides irrefutable evidence for how strategies that favored now illegal discriminatory practices helped to establish and maintain the current inequities that exist in academic medicine (60). Redistributing resources and codifying justice into programs aimed at overcoming historical and ongoing inequities are essential to attaining indemnity and providing restitution for both past and present racist and discriminatory practices by academic medicine and academic health centers. Beyond reversing “underrepresentation,” these interventions may mitigate some elements of structural racism within the healthcare system currently acting as drivers that permit the ongoing disparate loss of lives for those from minoritized and marginalized communities.

## Conclusion

The enduring challenge for academic medicine is remaining intentionally invested and engaged in successfully eliminating the factors contributing to attrition of URiM students, trainees and physician scientists. Focused commitment and preparation must accompany plans for restitution through equity-focused mentoring. Medical schools and academic health centers need to guarantee unwavering support of pathways to promotion and tenure that champion activities focused on mentoring and professional development of URiM physicians (58). This includes institutional budgets that designate financial and administrative support, e.g., valued time for mentoring activities at a scale comparable to metrics defined for RVUs. Assigning explicit value for the efforts required to achieve equity-focused mentoring can assist in decreasing risk of burnout and attrition among BLNA faculty.

At the institutional level, incorporating a mentoring curriculum and training for faculty that increases cultural intelligence, demands accountability for performance, and grants continuing education credit can serve as a required component of professional development metrics (57–59). Instituting these changes moves organizations from statements of solidarity to standards for solidarity. Educating and training well-informed institutional leaders to act as allies for enacting policy changes that truly foster a more diverse, equitable and inclusive work environment builds on the success of these mentoring curricula. It also represents the next generation of accountability metrics. Another critical competency requires planning and preparation for the backlash that inevitably labels these efforts as unnecessary or indicative of reverse discrimination (60).

When considering the promises and perils of embracing equity-focused mentoring, “The Gardener's Tale” provides a cautionary tale. In the absence of a supportive, nurturing environment with thoughtful planting and pruning, flowers may not bloom or thrive. Mentors provide the rich soil of support and understanding where rocks of discrimination and exclusion once predominated. Mentors help to prevent premature pruning when blooming takes longer than usual or is less robust than expected. They add fertilization to the environment by imparting reassurance and a sense of belonging. Faculty involved in mentoring deserve meaningful valuation by their academic institution, and where possible, receive training and support to attain grant funding that researches equity-focused mentoring frameworks and models. Furthermore, pathways for career advancement need an explicit trajectory to achieving success for those faculty mentoring and empowering a future generation of diverse physicians and scientists. The road to equitable representation within the physician workforce must be paved with adequate resources and paid through financial restitution.

## Data Availability Statement

The original contributions presented in the study are included in the article/supplementary material, further inquiries can be directed to the corresponding author.

## Author Contributions

VW and DW developed the concept for the paper. VW wrote the first draft and made additional revisions and edits. DW made additional revisions and edits and performed additional literature review. All authors edited, reviewed, and finalized the manuscript prior to submission. All authors contributed to the article and approved the submitted version.

## Conflict of Interest

The authors declare that the research was conducted in the absence of any commercial or financial relationships that could be construed as a potential conflict of interest.

## Publisher's Note

All claims expressed in this article are solely those of the authors and do not necessarily represent those of their affiliated organizations, or those of the publisher, the editors and the reviewers. Any product that may be evaluated in this article, or claim that may be made by its manufacturer, is not guaranteed or endorsed by the publisher.
